# Characteristics of hemolytic activity induced by skin secretions of the frog *Kaloula pulchra hainana*

**DOI:** 10.1186/1678-9199-19-9

**Published:** 2013-04-18

**Authors:** Shuangshuang Wei, Tingting Chi, Aiyun Meng, Congwei Chen, Tianchen An, Manchuriga Wang, Yingxia Zhang

**Affiliations:** 1 Key Laboratory of Tropic Biological Resources, Minister of Education, College of Marine Science, Hainan University, Haikou, China; 2 College of Agriculture, Hainan University, 58, Renmin Road, Haikou, Hainan, 570228, China

**Keywords:** Amphibian, Skin secretions, Hemolysis, Pore-forming

## Abstract

**Background:**

The hemolytic activity of skin secretions obtained by stimulating the frog *Kaloula pulchra hainana* with diethyl ether was tested using human, cattle, rabbit, and chicken erythrocytes. The skin secretions had a significant concentration-dependent hemolytic effect on erythrocytes. The hemolytic activity of the skin secretions was studied in the presence of osmotic protectants (polyethylene glycols and carbohydrates), cations (Mg^2+^, Ca^2+^, Ba^2+^, Cu^2+^, and K^+^), or antioxidants (ascorbic acid, reduced glutathione, and cysteine).

**Results:**

Depending on their molecular mass, osmotic protectants effectively inhibited hemolysis. The inhibition of skin hemolysis was observed after treatment with polyethylene glycols (1000, 3400, and 6000 Da). Among divalent cations, only 1 mM Cu^2+^ markedly inhibited hemolytic activity. Antioxidant compounds slightly reduced the hemolytic activity.

**Conclusions:**

The results suggested that skin secretions of *K. pulchra hainana* induce a pore-forming mechanism to form pores with a diameter of 1.36-2.0 nm rather than causing oxidative damage to the erythrocyte membrane.

## Background

The skin of amphibians has numerous biochemical and physiological functions to assure amphibian survival. Several bioactive components with specialized functions and molecular structures have been isolated and purified from amphibian skin [1,2]. Some of these components and their analogs have been used for treating diseases such as microbial infections and burns [1,3]. Thus, increasing numbers of studies have focused on amphibian skin secretions to identify biologically active proteins and peptides [1,4].

Cnidarian venom is an abundant source of numerous bioactive molecules such as pore-forming proteins, small cytotoxic peptides, 5-hydroxytryptamine, and histamine. Of the cytolytic, hemolytic, and neurotoxic effects of Cnidarian venom, hemolytic activity is the most commonly investigated [5-7]. Hemolysis induced by Cnidarian venom toxins has been particularly investigated to identify targets and the attachment of proteins to cell membranes [8,9].

*Kaloula pulchra hainana* (Anura) is an endemic amphibian found in the low elevation regions of Hainan Island of China. These frogs always live near pools, which is a harsh environment with numerous pathogens. When these frogs are stimulated, their belly bulges and white secretion is released from their skin. In a previous study, we purified and characterized a 23-kDa trypsin inhibitor from the skin secretions of *K. pulchra hainana*, designated *K. pulchra hainana* trypsin inhibitor (KPHTI) [10].

Skin secretions of the frog *K. pulchra hainana* also exhibit hemolytic activity. This study aimed to establish basic information on erythrocyte hemolysis and cell membrane peroxidation induced by these skin secretions. To determine the toxicological properties of the skin secretions, we investigated the effects of different factors on erythrocyte hemolysis, including osmotic protectants, cations, antioxidants, and chelating agents.

## Methods

### Materials

N-acetyl-D-galactosamine (NAGA), N-methylmannopyranose, D-glucose, D-trehalose, D-lactose, reduced glutathione (GSH), and bovine serum albumin (BSA) were purchased from Sigma-Aldrich (USA). Polyethylene glycols (PEGs) of different molecular masses (300, 400, 1000, 3400, and 6000 Da) were obtained from Fluka (USA). The protein concentrations of skin secretions were determined using a protein assay kit (Bio-Rad, USA) with BSA as a standard [11]. All other reagents used were of the highest purity available.

### Preparation of skin secretions

Adult specimens of *K. pulchra hainana* of both genders (n = 10; weight range: 80–120 g) were collected in Danzhou City, Hainan Province in southern China. The skin of the frogs was briefly stimulated with diethyl ether and then washed with 50 mM Tris–HCl buffer (pH 7.8) containing 0.1 M NaCl and 5 mM ethylene diamine tetraacetic acid (EDTA). The secretions were centrifuged at 10,000 g for 20 minutes at 4°C to remove insoluble materials. The supernatant was collected, lyophilized, and stored at −80°C until use. Before an experiment, the lyophilized skin secretions were dissolved in phosphate-buffered saline (PBS) (137 mM NaCl, 1.5 mM KH_2_PO_4_, 2.7 mM KCl, 8.1 mM Na_2_HPO_4_) and then dialyzed against PBS.

### Hemolysis determinations

The hemolytic activity of skin secretions was determined using human, cattle, rabbit, and chicken erythrocytes, as reported by Liu *et al*. [12]. Erythrocytes from these species were washed with PBS until the supernatant was clear and then resuspended in PBS. Erythrocyte suspensions (5 × 10^6^ cells/mL) were incubated with different concentrations of skin secretions (0.28, 0.56, 1.4, 2.8, 4.2, and 5.6 μg/mL) at 37°C for 30 minutes and then centrifuged at 1000 g for 5 minutes at 4°C to precipitate intact erythrocytes and debris. The supernatants were assayed for absorbance at 540 nm to determine the percentage of hemoglobin released from the lysed erythrocytes. We defined 100% lysis as the absorbance of a supernatant obtained using 1% Triton X-100 instead of test samples. The hemolytic activity of the skin secretions was expressed as the percentage of absorbance compared with that observed after 100% lysis induced by Triton X-100. The supernatant of an untreated erythrocyte suspension in PBS was used as a spectrophotometric blank.

### Determination of membrane pore diameters

The diameters of membrane pores induced by skin secretions were determined as described previously [13]. In brief, 40 mOsm PEGs of various molecular masses (300, 400, 1000, 3400, and 6000 Da) were added to PBS to counteract the osmotic pressure of hemoglobin [14]. Following this, by changing the concentration of NaCl, the total osmotic pressure of the extracellular fluid was adjusted to 295 mOsm. After human erythrocytes were suspended in a PEG solution (5 × 10^6^ cells/mL), the hemolytic activity of skin secretions was determined as described above. The hydrodynamic diameters of PEG 300, 400, 1000, 3400, and 6000 were 1.16, 1.36, 2.0, 3.8, and 5.8 nm, respectively [13,15].

### Osmotic protectants

The following osmotic protectants were used: 5 mM NAGA, 10 mM N-methylmannopyranose, 25 mM D-glucose, 25 mM D-trehalose, and 25 mM D-lactose. Each of these was added to erythrocytes suspended in PBS. After skin secretions (5.6 μg/mL) were added and incubated at 37°C for 30 minutes, the hemolytic activity was determined as described above. For osmotic protectants with large molecular masses, erythrocytes were preincubated with PEG 6000 for 10 minutes, following which erythrocytes were removed and resuspended in PBS. Following this, skin secretions (0.7, 1.4, or 5.6 μg/mL) were added and hemolytic activity was determined.

### Cations and EDTA

Different cations (KCl, BaCl_2_, MgCl_2_, CuSO_4_, or CaCl_2_) were individually mixed with erythrocyte suspensions, following which skin secretions (1.4 or 5.6 μg/mL) were added and incubated at 37°C for 30 minutes and hemolytic activity was determined. The final concentration of CaCl_2_, BaCl_2_, MgCl_2_, and KCl was 10 mM, whereas that of CuSO_4_ was 1 mM. The effect of EDTA (0.1 mM, 0.2 mM, or 1 mM) was also determined.

### Antioxidants

Ascorbic acid, GSH, and cysteine were used to assess possible erythrocyte cell membrane oxidative damage induced by skin secretions. An antioxidant (2 mM) was added to an erythrocyte suspension, following which skin secretions were added (5.6 μg/mL) and hemolytic activity was determined.

### Statistical analysis

Results are given as means ± standard error (SE) for ten experiments. Results for different experimental conditions were compared by Student’s t-tests. A p-value < 0.05 was considered significant [16].

## Results

### Hemolytic activity of *K. Pulchra hainana* skin secretions

**Figure 1:**
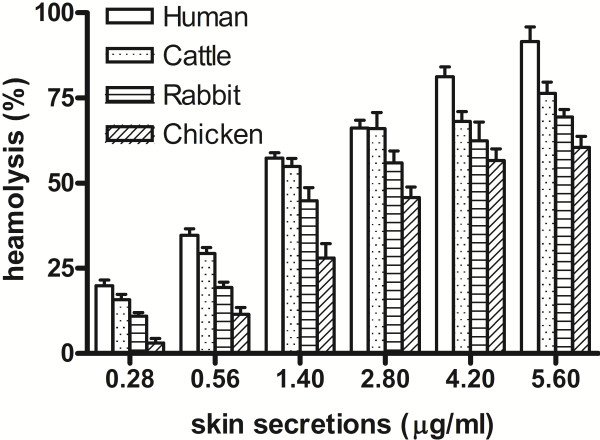
**Hemolytic activity of skin secretions from *Kaloula pulchra hainana *tested with erythrocytes from different species: human (□), cattle (Δ), rabbit (○), and chicken (▼ ).**

The frog skin secretions induced the hemolysis of erythrocytes from different species in a dose-dependent manner (Figure 1). The sensitivities of these erythrocytes to the secretions were different; the most sensitive erythrocytes were human erythrocytes. For human erythrocytes, the percent hemolysis was 19.8 ± 3% with 0.28 μg/mL of skin secretions, while total hemolysis was 90.3 ± 4% with 5.6 μg/mL of skin secretions. Among the four species examined, chicken erythrocytes were the least susceptible to these skin secretions; the hemolytic activity was approximately 60.5 ± 2% with the highest concentration of skin secretions (5.6 μg/mL).

**Figure 2:**
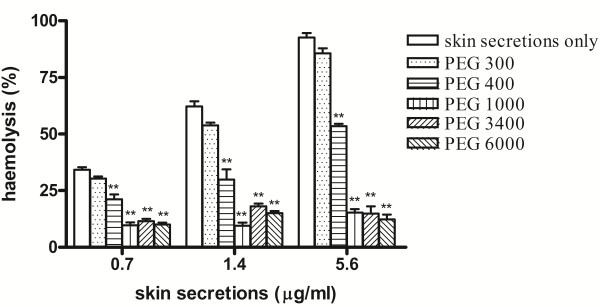
**Osmotic protection against hemolytic activity using a series of polyethylenglycols (PEGs; 300, 400, 1000, 3400, and 6000 Da).** Aliquots of skin secretions from *Kaloula pulchra hainana* (0.7, 1.4, or 5.6 μg/mL) were added to erythrocyte suspensions containing PEGs at a final concentration of 25 mM (n = 10). **p < 0.01.

The hemolytic activity of the skin secretions was tested in the presence of PEGs with different hydrodynamic diameters: 300, 400, 1000, 3400, and 6000 Da. These were used to estimate pore diameters in erythrocyte cell membranes. Hemolysis induced by the skin secretions was not affected by treatment with PEG 300, it was partially inhibited by PEG 400, and markedly inhibited by treatment with PEGs 1000, 3400, and 6000 (p < 0.05, compared with no PEGs; Figure 2).

### Effects of osmotic protectants on hemolytic activity

Erythrocyte suspensions were first pretreated with 25 mM PEG 6000 for 5 minutes, following which erythrocytes were removed and resuspended in PBS. Following this, skin secretions (0.7, 1.4, or 5.6 μg/mL) were added to test their hemolytic activities. The erythrocytes were hemolyzed equally compared with controls, which supported that the larger PEG molecules did not bind to the membrane to reduce the interaction between the cell membrane and skin secretions. However, because PEG 6000 is an osmotic protectant, it maintained the medium as more hypertonic and blocked membrane pores induced by the skin secretions.

Compared with the controls, the osmotic protectants with small molecular masses, including NAGA, D-glucose, methylmannopyranose, trehalose, and lactose, did not significantly inhibit the hemolytic activity of the skin secretions (Table 1).

**Table 1:** **Effect of different agents on the hemolytic activity of skin secretions of *Kaloula pulchra hainana***

**Agent**	**Inhibition (%)**
*Carbohydrates*	
D-glucose (25 mM)	12.1
D-trehalose (25 mM)	15.6
D-lactose (25 mM)	11.9
N-methylmannopyranose (10 mM)	6.9
N-acetyl-D-galactosamine (10 mM)	11.7
*Antioxidants*	
GSH (2 mM)	20.9
Cysteine (2 mM)	14.3
Ascorbic acid (2 mM)	14.7
*Chelator*	
EDTA (0.1 mM)	5
EDTA (0.2 mM)	7.3
EDTA (2 mM)	10

Hemolytic activity was assessed using skin secretions with a final concentration of 5.6 μg/mL. Percent inhibition with respect to the control is the mean of 10 experiments.

### Effects of cations and EDTA on hemolytic activity

**Figure 3:**
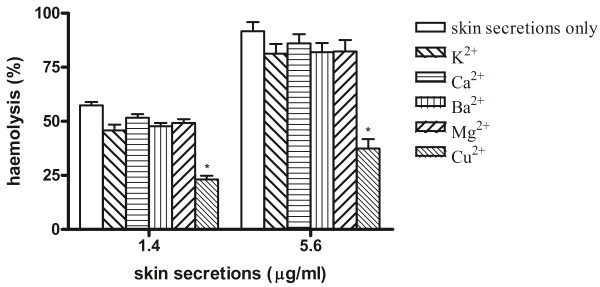
**Effects of cations (10 mM Ca**^**2+**^**, 10 mM Ba**^**2+**^**, 10 mM Mg**^**2+**^**, 1 mM Cu**^**2+**^**, and 10 mM K**^**+**^**) on the hemolytic activity of skin secretions of *Kaloula pulchra hainana*.** Each cation was incubated with erythrocyte suspensions, following which 1.4 or 5.6 μg/mL of skin secretions was added (n = 10), *p < 0.05.

The effects of different cations on the hemolytic activity of the skin secretions were assessed using the divalent cations Ca^2+^, Mg^2+^, Ba^2+^, and Cu^2+^ and the monovalent cation K^+^. Except for Cu^2+^, these cations at concentrations of < 10 mM did not affect the hemolytic activity compared with the control (data not shown). Cu^2+^ at a concentration of 1 mM significantly inhibited the hemolytic activity at skin secretion concentrations of 1.4 and 5.6 μg/mL, whereas 10 mM Mg^2+^, Ca^2+^, and K^+^ had slight inhibitory effects (Figure 3). EDTA at any of the concentrations used did not inhibit hemolysis (Table 1).

### Effects of antioxidants on hemolytic activity

The antioxidants cysteine, GSH, and ascorbic acid (2 mM) reduced the hemolysis induced by skin secretions, with results ranging from 14 to 20% (Table 1). However, these results were not significantly different from those of controls.

## Discussion

Amphibian skin is a convenient source of substantial amounts of granular gland secretions from which numerous components with specialized functions have been isolated and characterized [1,2,17,18]. In a previous study, we found that the skin secretions from *K. pulchra hainana* exhibited diverse biological activities, including protease inhibitory, cytotoxic, and hemolytic activities [10].

Hemolysis can be induced by several protein toxins from animals, plants, and microbes, particularly marine animals [19]. Some of these venoms affect biological membranes by inducing the formation of pores or channels in natural and model bilayer lipid membranes [20-22]. Thus, hemolytic activity induced by protein toxins has been used as a sensitive toxicological tool to investigate the targeting and attachment of proteins to cell membranes [15].

We used a series of PEGs (300, 400, 1000, 3400, and 6000 Da) with different hydrodynamic diameters to determine their effects on erythrocyte hemolysis induced by the skin secretions of *K. pulchra hainana*[15,23]. The small polymers PEG 300 did not affect hemolysis, while PEG 400 only partially inhibited hemolysis. However, hemolysis was significantly inhibited by treatment with larger PEGs of 1000, 3400, and 6000 Da (Figure 2). We deduced that these skin secretions had induced the formation of transmembrane pores in erythrocyte membranes. In contrast, small osmolytes, including glucose, trehalose, lactose, methylmanopyranose, and galactosamine, did not affect hemolysis (Table 1). Thus, we hypothesize that the hydrophilic pores in erythrocyte cell membranes induced by these skin secretions caused a colloid osmotic burst that resulted in erythrocyte lysis. The diameters of these pores were approximately 1.36-2.0 nm based on the hydrodynamic diameters of PEG 400 and 1000 of 1.36 nm and 2.0 nm, respectively [24].

In addition to pore-forming mechanisms, lipid peroxidation of erythrocyte membranes plays an important role in the hemolysis induced by hemolytic protein toxins, resulting in cell membrane disorder [19,20]. The antioxidant compounds GSH, cysteine, and ascorbic acid only minimally reduced the hemolytic activity of these skin secretions. From these results, we deduced that these skin secretions induced erythrocyte lysis by inducing pore formation in bilayer lipid membranes rather than causing oxidative damage.

βγ-CAT, a protein purified from the skin secretions of the frog *Bombina maxima*, has potent hemolytic activity and induces the formation of membrane pores with diameters of approximately 2.0 nm [12]. Antimicrobial peptides from amphibian skin also exhibit hemolytic activity. In that report, the antimicrobial activity of the skin secretions was not detected for some gram-positive bacteria and gram-negative bacteria, suggesting that hemolysis was not caused by antimicrobial peptides. Preincubating these skin secretions with trypsin or collagenase resulted in a significant loss of hemolytic activity (unpublished observations). Proteins in *K. pulchra hainana* skin secretions were analyzed by sodium dodecyl sulfate polyacrylamide gel electrophoresis (SDS-PAGE). Two major bands were identified with molecular weights of approximately 28–60 kDa and 17–25 kDa (unpublished observations). However, we have no data regarding which proteins contributed to the hemolytic activity of these skin secretions.

Cations, particularly Ca^2+^, affect the hemolytic activities of sea anemone toxins, although different results are obtained depending on the specimens and toxin structures [25-27]. In our study, the hemolytic activity of skin secretions was slightly affected by 10 mM Ca^2+^, whereas it was significantly inhibited by 1 mM Cu^2+^. The chelating agent EDTA at any tested concentration did not produce any effects, suggesting that cations were not necessary for the hemolytic activity of these skin secretions.

## Conclusion

In this study, we analyzed the effects of osmotic protectants, cations, and antioxidants on erythrocyte hemolysis induced by the skin secretions from the frog *K. pulchra hainana*. We observed that osmotic protectants of high molecular mass inhibited this hemolytic activity. Cu^2+^ also significantly inhibited the hemolytic activity. We deduced that these skin secretions induced erythrocytes lysis by a pore-forming mechanism in the bilayer lipid membrane. This hypothesis needs to be explored in detail in future investigations.

## Abbreviations

PEGs: Polyethylene glycols; NAGA: N-acetyl-D-galactosamine; GSH: Reduced glutathione; BSA: Bovine serum albumin; EDTA: Ethylene diamine tetraacetic acid; KPHTI: *Kaloula pulchra hainana* trypsin inhibitor; PBS: Phosphate-buffered saline; SE: Standard error; SDS-PAGE: Sodium dodecyl sulfate polyacrylamide gel electrophoresis.

## Competing interests

The authors declare that they have no competing interests.

## Authors’ contributions

ZYX, WSS, CTT, MAY conceived and designed the experiments, and wrote the paper. WSS, CTT, MAY, CCW, ATC, Wang M have made substantial contributions to acquisition of data, and analysis and interpretation of data. All authors have given final approval of the version to be published.

## Financial source

This work was supported by the grants from the National Natural Science Foundation (30700128, 30960053), Natural Science Foundation of Hainan Province (312067), and Foundation of Hainan University (hd09xm67qnjj1148).
